# HIV-1 CRF07_BC transmission dynamics in China: two decades of national molecular surveillance

**DOI:** 10.1080/22221751.2021.1978822

**Published:** 2021-09-27

**Authors:** Zhangwen Ge, Yi Feng, Hua Zhang, Abdur Rashid, Silvere D. Zaongo, Kang Li, Yueyang Yu, Bowen Lv, Jia Sun, Yanling Liang, Hui Xing, Anders Sönnerborg, Ping Ma, Yiming Shao

**Affiliations:** a State Key Laboratory for Infectious Disease Prevention and Control, National Center for AIDS/STD Control and Prevention, Chinese Center for Disease Control and Prevention, Beijing, People’s Republic of China; b Department of Laboratory Medicine, Guizhou Provincial People’s Hospital, Affiliated Hospital of Guizhou University, Guiyang, People’s Republic of China; c School of Medicine, Nankai University, Tianjin, People’s Republic of China; d Department of Infectious Diseases, Nankai University Second People’s Hospital, Tianjin Second People’s Hospital, Tianjin, People’s Republic of China; e State Key Laboratory for Diagnosis and Treatment of Infectious Diseases, Collaborative Innovation Center for Diagnosis and Treatment of Infectious Diseases, The First Affiliated Hospital, School of Medicine, Zhejiang University, Hangzhou, People’s Republic of China; f Division of Clinical Microbiology, Department of Laboratory Medicine, Karolinska Institutet, Stockholm, Sweden

**Keywords:** HIV-1, CRF07_BC, China, molecular epidemiology, surveillance, transmission cluster, risk factors

## Abstract

By analyzing an unprecedentedly large, longitudinal HIV-1 CRF07_BC sequence dataset collected from China in the past two decades, we sought to build CRF07_BC lengthwise transmission networks, and understand its transmission dynamics. We divided CRF07_BC into two clusters based on phylogenetic analysis and an estimation of the pairwise genetic distance at 0.7%. Of 6213 sequences, 3607 (58.1%) linked to ≥1 other sequence. CRF07_BC was divided into two clusters: 07BC_O and 07BC_N. The 07BC_O is the original CRF07_BC, circulating in people who inject drugs (PWID) and heterosexuals, predominantly in southwestern and northwestern provinces of China. The 07BC_N is a new cluster, identified mostly in men having sex with men (MSM) in the northern provinces of China. Bayesian analysis indicates that CRF07_BC has experienced two phases of exponential growth, which was first driven by 07BC_O then 07BC_N. Compared to 07BC_O, the proportion of the parameter of population transmission risk (TR) of 07BC_N has risen constantly. The power-law function analyses reveal that 07BC_N has increased over years with higher degree. In 07BC_N, only 13.16% of MSM were linked to other risk groups, but these links represent 41.45%, 54.25%, and 55.07% of links among heterosexual females, heterosexual males, and male PWID respectively. This study indicates that CRF07_BC has evolved into two clusters in China, and their distributions are distinct across risk groups and geographical regions. 07BC_N shows a greater risk of transmission, and has gradually replaced 07BC_O. Furthermore, the results show that strengthening the MSM interventions could lower the rapidity of 07BC_N transmission in all risk groups.

## Introduction

The CRF07_BC is a variant of a recombination of the HIV-1 B’ and C among people who inject drugs (PWID) in southwest China in the 1990s [1]. The CRF07_BC had spread over PWIDs through the drug trafficking route to Xinjiang in northwest China and then to other parts of the country both over PWIDs and heterosexuals [1]. During the twenty-first century, since a stringent campaign was launched to control drug trafficking and drug abuse, and implement methadone maintenance treatment programmes [2], CRF07_BC has spread more and more frequently through sexual contacts, both among heterosexual and male homosexual populations [3,4].

According to the latest nationwide HIV-1 molecular epidemiological survey in 2018 [5], CRF07_BC has become the most prevalent strain in China. Moreover, a series of surveys in various provinces suggest that CRF07_BC had played an essential role in HIV-1 transmission among men who have sex with men (MSM) [6,7]. CRF07_BC strains were frequently detected in MSM in some parts of China [7–9] in recent years. However, a detailed tracking of its transmission patterns across populations and regions is impossible without mapping the spread at the nationwide level.

The huge number of HIV-infected population in China has been causing much pressure on HIV prevention and control [10]. With the increased burden of case management, innovative techniques are in urgent need to reduce secondary transmission of HIV and control the aggravating spread to more populations [10,11]. Techniques for reconstructing HIV-1 transmission networks from HIV-1 sequence database enable an estimate of the unobserved transmission network [12]. Previous studies have investigated the demographic and risk characteristics of HIV-1 transmission networks [13] and building transmission networks have been proven to be a powerful tool in intervention policy by many studies [14]. This study constructs longitudinal transmission networks with Chinese HIV-1 CRF07_BC strain sequences database obtained from 1997 to 2017 at the nationwide level, and also provides insights into the depth of CRF07_BC transmission among different risk groups. Moreover, new parameters were used to assess the transmission dynamics of the strain over the dataset period. The results provide insights into the temporal and spatial transmission characteristics of HIV-1 CRF07_BC. In terms of practical implications, we propose several accurate intervention strategies to policy-makers for disease control.

## Materials and methods

### Data collection

The sequences of 5099 patients for the nationwide cross-sectional HIV-1 molecular epidemiological surveys prior to 2017 were extracted from the National Center for AIDS/STD Control and Prevention (NCAIDS). Sampling locations were grouped into seven regions (Supplementary Figure S1) according to their proximity and socio-economic status in accordance with the guidelines from the National HIV-1 Surveillance system of China. Transmission categories include MSM, PWID (stratified as male PWID and female PWID), and heterosexuals (separated as heterosexual males and heterosexual females). The missing data in transmission categories were designated as unknown. Sequences from NCAIDS have not been uploaded to any databases (e.g. GenBank) and only one sequence (the earliest) per individual was included.

Thereafter, additional 1114 HIV-1 CRF07_BC sequences, which were sampled in China prior to 2017, were collected from Los Alamos HIV-1 sequence database (http://www.HIV.lanl.gov). Only one sequence (the earliest or the longest) from the same individual was included. The methods are illustrated step by step in Supplementary Figure S2.

The viral sequences containing ≥5% ambiguities were excluded. The sequences less than 920 nucleotides in length were also excluded. Eventually, 6213 sequences (*pol*, HXB2 coordinates: 2253–3467 bp) – associated with information such as sampling year, transmission route, gender, age at diagnosis, provinces, and marital status – were included. The study was approved by the institutional review boards of NCAIDS, China CDC.

### Sequence analysis and transmission networks construction

Based on the collected nucleotide sequences, a maximum likelihood phylogenetic tree was built using FastTree 2.3 software, with a GTR nucleotide substitution model to confirm the genotypes and phylogenetic clusters. Then, we calculated the genetic distance (TN93) for all pairs of sequences identified as CRF07-BC using the HyPhy package. The network was constructed under the threshold of 0.7% genetic distance by linking two individuals (nodes) using cytoscape_v3.7.0. CRF07_BC was divided into two clusters in the transmission networks when the gene distance was 0.7%: 07BC_O and 07BC_N. We described the characteristics of the network including the number of sequences (nodes), links (degrees/edges), and clusters (groups of linked sequences) [13]. The degree of each individual was defined as the number of edges linked to other individuals.

### Comparison of clusters 07BC_N and 07BC_O

We compared the two clusters from the number of individuals, links, links/individuals, and the largest cluster in cumulative transmission networks (CTNs).

Individuals of each CTNs were divided into three population groups by quartiles according to degrees which were defined as low-level (0 link, two quartiles of the population), medium-level (1–5 links, one quartile of the population), and high-level (≥6 links, the highest quartile of the population). To evaluate the change of transmission risk between 07BC_N and 07BC_O under the different levels of CTNs, we put forward the parameter of population transmission risk (TR). TR of one cluster was defined as the sum of the degrees of all individuals of this group for medium-level and high-level, and the number of individuals for low-level. Then, we calculated constituent ratios of TR of different clusters under each of the three levels from 1997–2008, 2009–2010, 2011–2012, 2013–2014, to 2015–2017 transmission networks.

The degree distribution of a network is a long-tailed distribution, which indicates that a large majority of nodes have a low degree but a small number have a high degree [15,16]. Such a distribution approximately follows a power-law distribution of which the general form of function can be represented as *y* = cx^(−*r*). To compare the trends of the degree distributions in 07BC_N and 07BC_O, their degree distributions from 1997–2008, 2009–2010, 2011–2012, 2013–2014, to 2015–2017 transmission networks were fitted into curves of power-law functions respectively.

### Bayesian demographic reconstruction of HIV-1 CRF07_BC

Because the number of sequences sampled from each province does not correlate well with the estimated number of new CRF07_BC infections in each province in 2015 (as determined by the 2015 nationwide cross-sectional survey), we randomly sampled sequences according to the proportion of CRF07_BC in each province in the 2015 nationwide cross-sectional survey data. To retain the full early samples, all samples prior to 2007 were retained in the subsets. In order to retain the full geographic distribution, provinces with less than 25 sequences available were not subsampled. Finally, a subset of 993 and 645 sequences were sampled from 07BC_N and 07BC_O datasets, respectively.

TempEst was used to validate that there were sufficient temporal signals in the subsets to estimate molecular clock phylogenies. Time phylogenies for the 07BC_N and 07BC_O subsets were constructed in BEAST 1.8.4, using an uncorrelated log-normal relaxed clock, a Bayesian skygrid coalescent model, and a GTR+ G4 substitution model [17,18].

### Analysis between sub-populations

To understand the extent to which HIV-1 transmission occurs between individuals of the same or different transmission categories of 07BC_O and 07BC_N, the characteristics of potential transmission partners were analysed. To account for the bias arising from individuals with multiple links, we followed the protocol outlined by Oster and colleagues [13]. Each potential transmission partner for an individual is weighted based on the number of links associated with the individual(n). The weight is simply 1/n for each of the n links.

### Statistical analysis

Univariate and multivariable logistic regressions were used to evaluate the factors associated with clustering in the network compared to the entire case with a genotype. All analyses were conducted in SPSS 22.0 software package and *p* values<0.05 were considered as significant.

## Results

### Characteristics of data and identification of two distinct HIV-1 CRF07_BC clusters in China

Overall, 6213 HIV-1 nucleotide sequences from 6213 persons were analysed. Among them, 3607 (58.06%) were in the network, 2946 (47.42%) were MSM, 5101 (81.10%) were male, 1702 (27.39%) were from the southwest, 2359 (37.97%) were aged 21–30 years old, and 1654 (26.62%) were unmarried (Table 1).

**Table 1.** Characteristics of study participants.

Attribute	Category	Total	07BC_N	07BC_O
	Total	6213	3597	2616
In/outside the network	In the network	3607	2607	1000
	Outside the network	2606	990	1616
Sampling year	1997–2008	535	90	445
	2009–2010	825	333	492
	2011–2012	977	619	358
	2013–2014	772	620	152
	2015–2017	3104	1935	1169
Risk groups	Heterosexual	2272	833	1439
	PWID	995	24	971
	MSM	2946	2740	206
Gender	Female	964	142	822
	Male	5101	3442	1659
	Unknown	148	13	135
Domicile	Central	386	300	86
	East	999	767	232
	North	1410	1138	272
	Northeast	126	105	21
	Northwest	662	137	525
	South	928	717	211
	Southwest	1702	433	1269
Age at diagnosis	≤20	407	260	147
	21–30	2359	1417	942
	31–40	937	435	502
	41–50	436	236	200
	51–60	225	101	124
	>60	199	52	147
	Unknown	1650	1096	554
Marital status	Married	1261	331	930
	Unmarried	1654	1153	501
	Divorced or widowed	322	133	189
	Unknown	2976	1980	996

Abrreviations: MSM, men who have sex with men; PWID, persons who use injection drugs.

**Figure 1.**
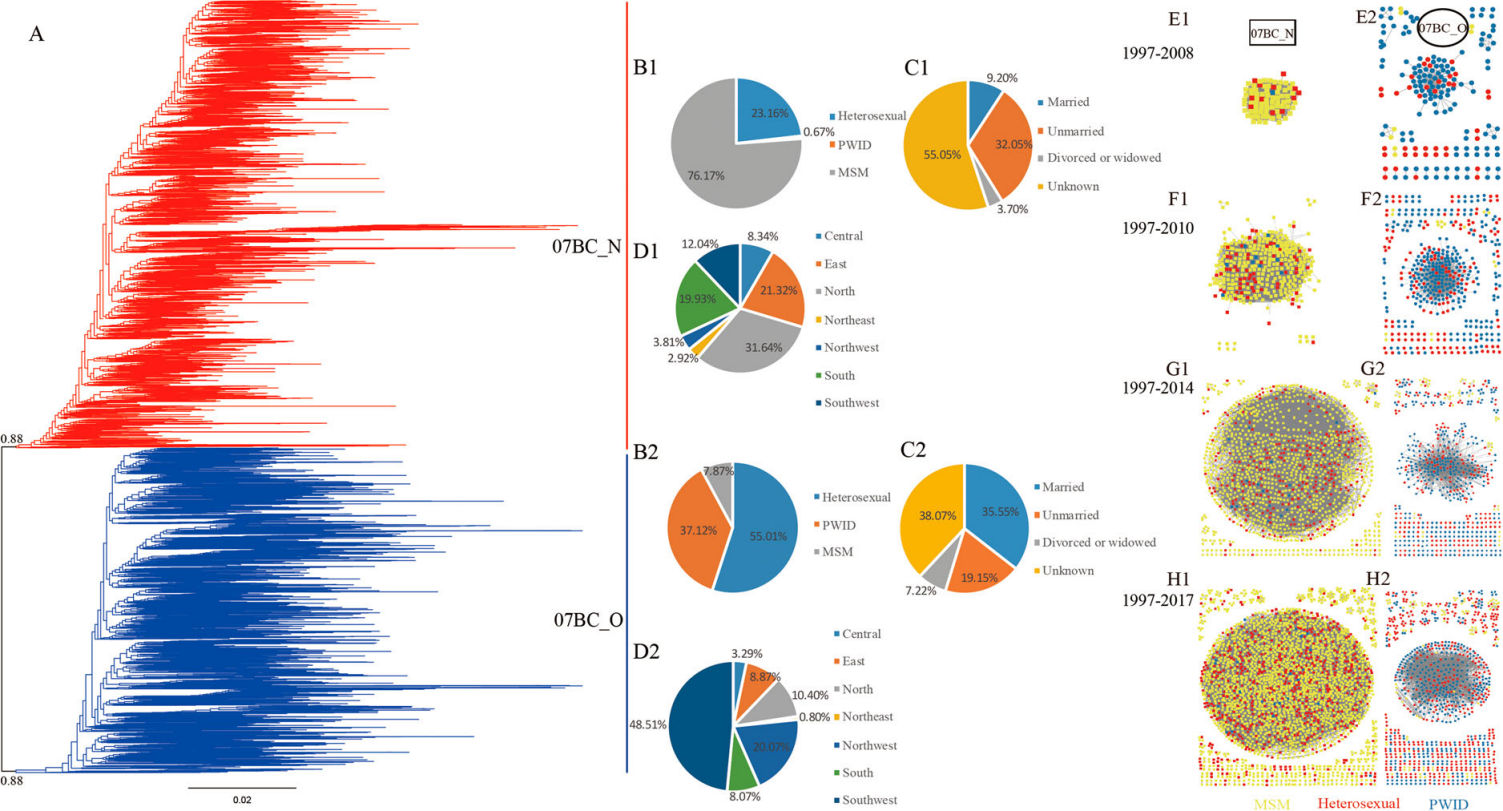
(Left) Maximum-likelihood phylogenetic trees of HIV-1 *pol* sequences from 6213 individuals (A). Red and blue lines represent study individuals of 07BC_N and 07BC_O respectively. (Middle) Distribution of individuals in two clusters among different populations. The proportion of individuals in 07BC_N among different transmission risk populations (B1), marital status populations (C1), and residential populations (D1). The proportion of individuals in 07BC_O among different transmission risk populations (B2), marital status populations (C2), residential populations (D2). (Right) Cumulative CRF07_BC transmission network from 1997 to 2008 (E1, E2), 2010 (F1, F2), 2014 (G1, G2) and 2017 (H1, H2). One node represents a sequence or an individual. An edge (link) represents the genetic distance (TN93) between connected sequences ≤ 0·007 substitutions/site. Only nodes connected with others within the network are shown. Edge lengths are optimized for visual presentation and do not represent genetic distance. The colour indicates transmission route. Square indicates 07BC_N on the left side of the image (E1, F1, G1, H1) and the circle indicates 07BC_O on the right (E2, F2, G2, H2). MSM: men who have sex with men; PWID, individuals who use injection drugs.

The maximum-likelihood phylogeny identified two well supported (bootstrap values > 85%) and distinct clusters of CRF07_BC strains (Figure 1(A)). Similar observations were made in the molecular network when the gene distance was 0.7% (Figure 1(E1,2; F1,2; G1,2; H1,2)). The new major transmission cluster (noted as 07BC_N) was predominant in MSM (76.17%), unmarried individuals (32.05%), and northern (31.64%) provinces of China (Figure 1(B1, C1, D1)). By contrast, another cluster (noted as 07BC_O) formed by original sequences mostly spread among heterosexual populations (55.01%), PWID (37.12%), married individuals (35.55%), with most cases reported in southwestern (48.51%), and northwestern (20.07%) provinces (Figure 1(B2, C2, D2)). Nevertheless, both 07BC_N and 07BC_O sequences have been sampled in all geographic regions of China and in all key populations (Table 1). Under the threshold of 0.7% genetic distance in CTNs from 1997 to 2008, 2010, 2014, and 2017: 75 (85.23%), 323 (80.15%), 1189 (81.44%), and 2202 (77.56%) MSM were respectively included for 07BC_N (Figure 1(E1, F1, G1, H1)); and 206 (95.37%), 477 (96.17%), 618 (91.83%), and 904 (90.4%) heterosexuals and PWID were respectively included for 07BC_O (Figure 1(E2, F2, G2, H2)). Across the study networks, the proportion of 07BC_N was 28.94% (in 1997–2008), 44.83% (in 1997–2010), 68.45% (in 1997–2014), and 72.28% (in 1997–2017) (Figure 1(E1,2; F1,2; G1,2; H1,2)); indicating that its proportion was increasing rapidly over time.

### Factors associated with clustering

The highest proportions of clustering were observed in 07BC_N (72.48%), the sampling year 2011–2014 (70.55%), MSM (71.89%), southern provinces (73.71%), and individuals below 20 years old (58.72%) (Supplementary Table S1). Some factors enhanced the odds of clustering significantly including 07BC_N (*p* < 0.001), sampling year during 1997–2010 (*p* < 0.001), MSM (*p* = 0.004), heterosexual males (*p* = 0.044), northern provinces (*p* = 0.044), and individuals over 60 years old (*p* = 0.016) (multivariable logistic regression; Supplementary Table S1).

### Trends of 07BC_N and 07BC_O parameters

**Figure 2.**
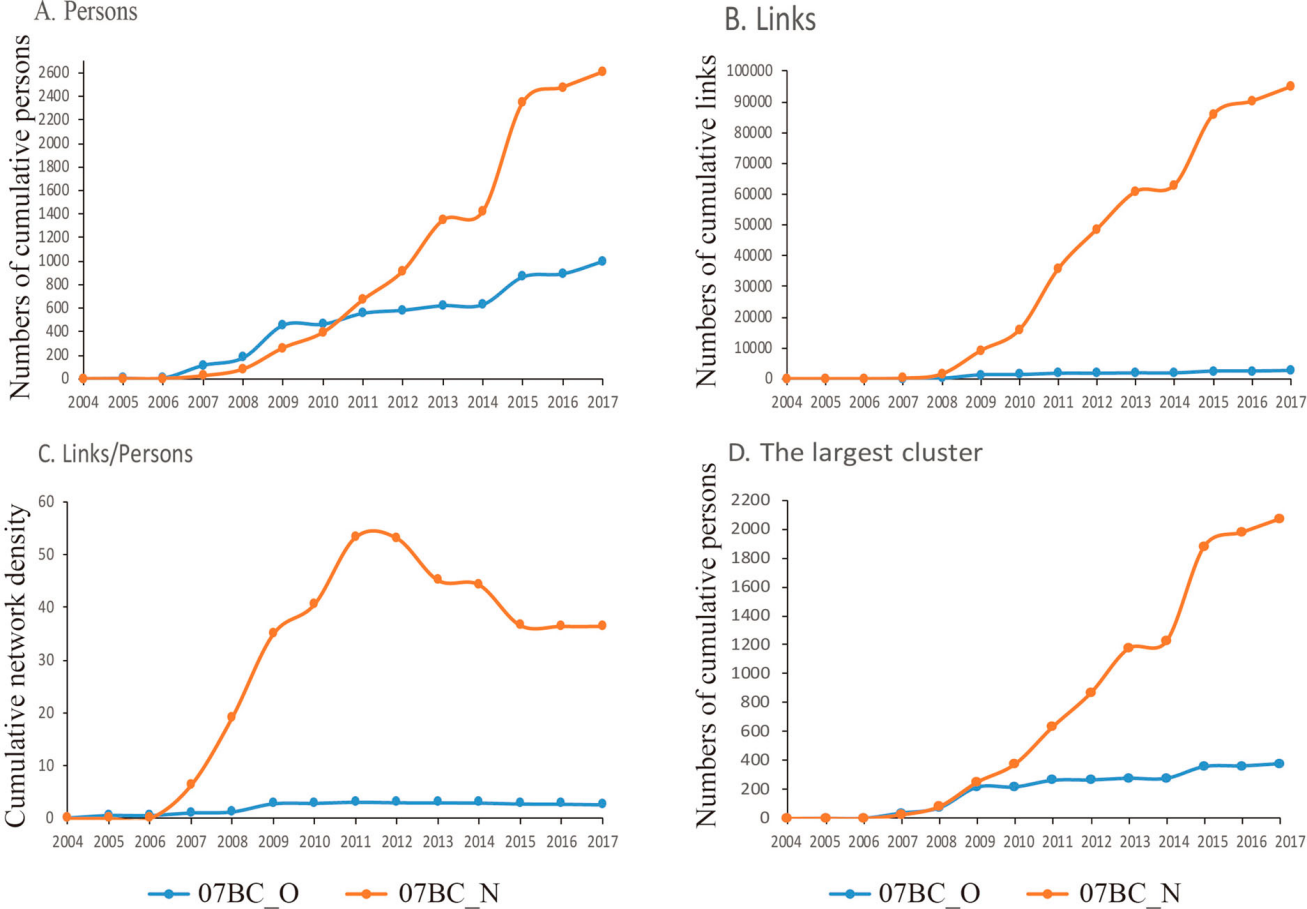
The number of individuals, links, links/individuals and the largest cluster in cumulative transmission networks (CTNs). The number of individuals (A), links (B), links/individuals (C) and the largest cluster (D) in CTNs marks on the y-axis and the sampling year is shown on the x-axis. Blue denotes 07BC_O and orange represents 07BC_N.

As shown in Figure 2, in the early stage, the numbers of individuals and links of 07BC_O were more than 07BC_N. However, the growth rate of 07BC_N showed a faster increase, and the numbers of individuals and links of 07BC_N have exceeded those of 07BC_O since 2011 and 2007 respectively (Figure 2(A, B)). The network density (links/individuals) of 07BC_N has exceeded 07BC_O since 2007 and reached the highest density in 2011 (Figure 2(C)). The numbers of individuals in the largest cluster for 07BC_N and 07BC_O showed a steady rising trend, though 07BC_N had a faster growth and the number of 07BC_N has exceeded the number of 07BC_O since 2008 (Figure 2(D)).

### Analysis of population transmission risk

**Figure 3.**
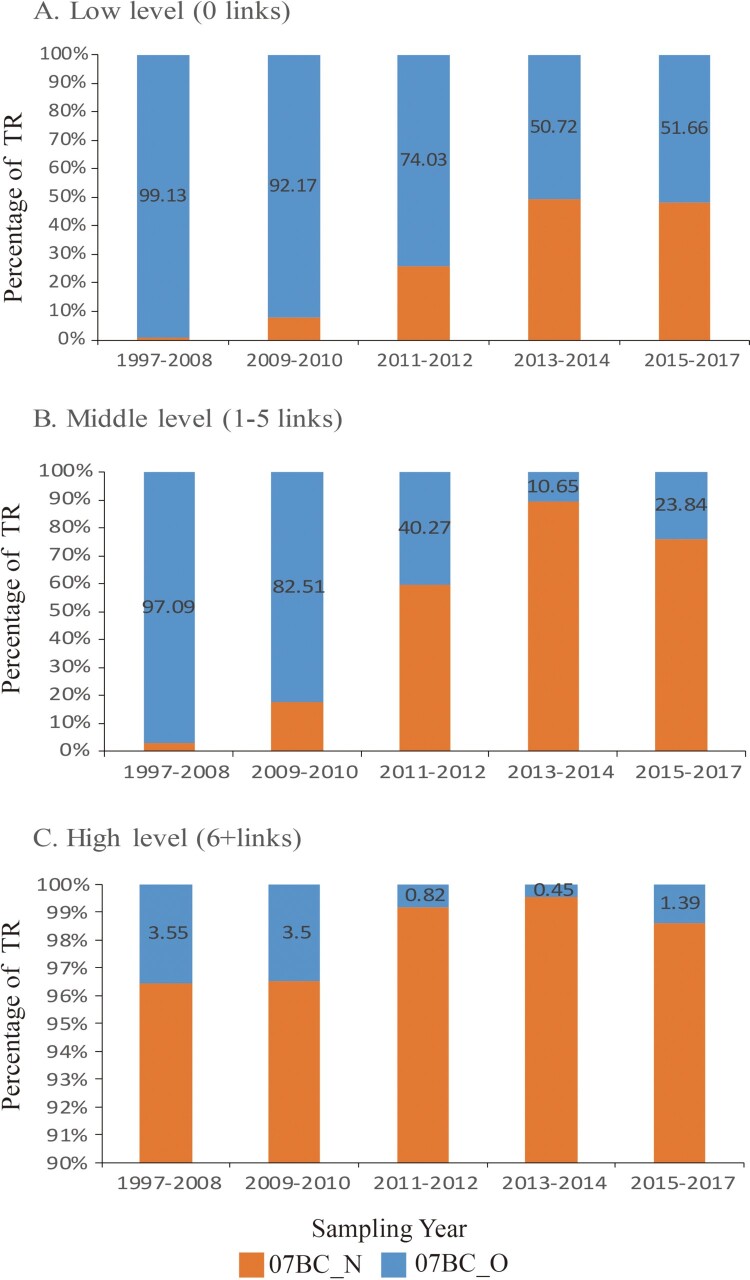
Trends of transmission risk parameter (TR) between two clusters. Each network was divided into three population groups according to quartiles degree. Low-level (two quartiles of the population): individuals linked to 0 other individuals. Medium-level (one quartile of the population): individuals linked to 1–5 other individuals. High-level (one quartile of the population): individuals linked to ≥6 other individuals. The sum of the degree of nodes for each cluster was calculated as the parameter of population transmission risk for medium-level and high-level, and the number of individuals as parameter of population transmission risk for low-level. The percentage of TR within two clusters marks on the y-axis and the sampling year is shown on x-axis. Colours correspond to clusters.

In the low-level population, the proportion of TR by 07BC_O was predominant, even though the percentage of 07BC_N rose gradually and went close to 07BC_O (49.34%) in 2017 (Figure 3(A)). In the medium-level population, the proportion of TR by 07BC_N increased from 2.91% to 76.16% and accounted for the majority since 2011 (Figure 3(B)). In the high-level population, 07BC_N represents more than 95% of TR since 1997–2008 and continued to rise (Figure 3(C)).

### The fitted power-law distribution of degree for 07BC_N and 07BC_O

**Figure 4.**
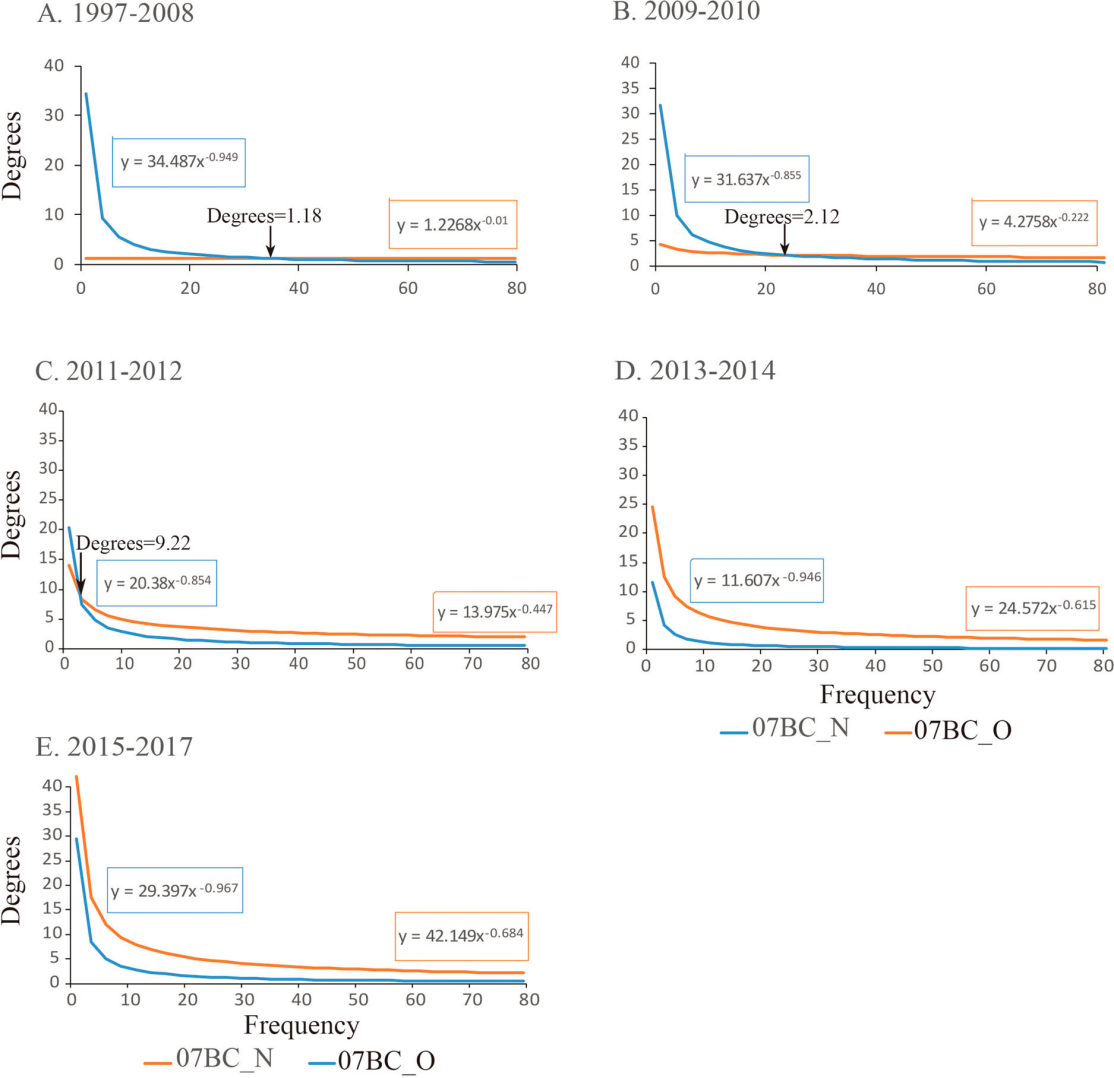
Power-law plot fitted from degree distribution between 07BC_N and 07BC_O in CRF07_BC networks. The graphs show the fitted distribution of degree among 07BC_N (blue) and 07BC_O (orange) in each CRF07_BC network. The fitted power-law function of the form y = cx^-r^ is shown. Besides, the intersection coordinates of two functions from 07BC_N (orange) and 07BC_O (blue) are shown.

Figure 4 illustrates the power-law curves fitted in 1997–2008, 2009–2010, 2011–2012, 2013–2014, and 2015–2017 transmission networks intersect. The cutoff degree was 1.18, 2.12, and 9.22 in 1997–2008, 2009–2010, and 2011–2012 respectively. In other words, in the 1997–2008, 2009–2010, and 2011–2012 transmission networks, the frequency of 07BC_O was more than 07BC_N among individuals with higher degree and less than 07BC_N among individuals with lower degree. No intersection showing in the curves after 2013 indicates that the frequency of 07BC_N has not been lower than 07BC_O regardless of degree value since 2013 (Figure 4(D,E)).

### Bayesian demographic reconstruction of HIV-1 CRF07_BC

**Figure 5.**
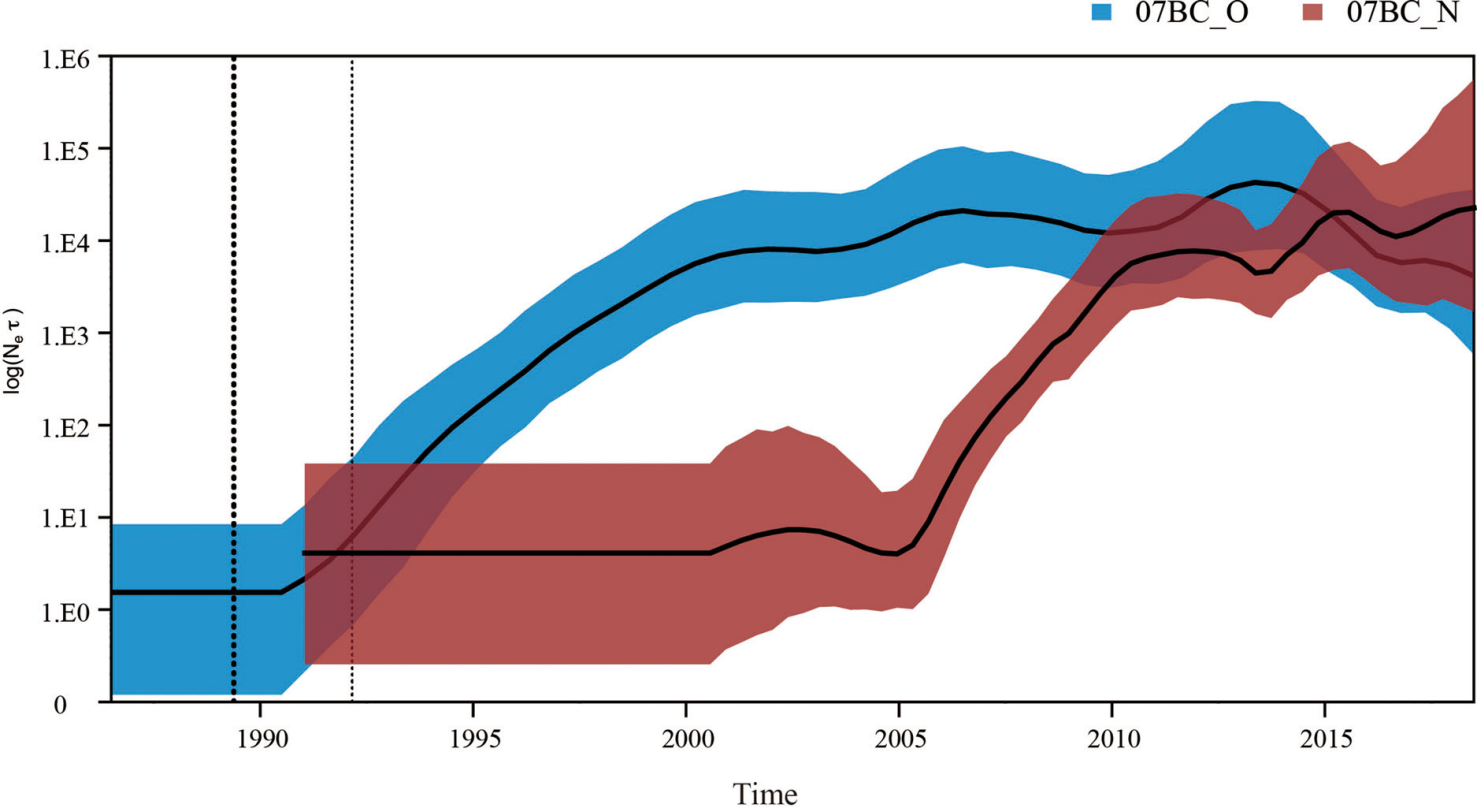
Bayesian skyline plot was estimated to reconstruct the demographic history of the two CRF07_BC clusters in China. The x-axis is the time in units of years and the y-axis is equal to the effective population size. The solid line and shaded region represent the median and 95% credibility interval of the effective population size through time. The demographic histories of the 07BC_O (blue) and 07BC_N (red) epidemics, as estimated by the Bayesian skygrid model, are based on subsets of 645 and 993 *pol* sequences respectively. While it appears that the epidemic among 07BC_O sequences reached its peak in the mid-2000s and is slowing down, all evidence points toward the 07BC_N epidemic still growing.

As shown in Figure 5, the Skyline plot result revealed that the past population dynamics of CRF07_BC had experienced two phases of exponential growth which were driven by 07BC_O and 07BC_N in turn: (1) 07BC_O had undergone a significant growth during 1991–2005 at an early stage and then a period of slightly declining population size, and (2) 07BC_N had a rapid growth after 2005 at the second stage. 07BC_N appeared late, expanded rapidly after 2005, gradually replaced 07BC_O, and became the largest cluster. Of note, after 2010, 07BC_O showed a declining trend, but 07BC_N still grew rapidly at high levels of prevalence.

### Transmission links between different transmission category

**Figure 6.**
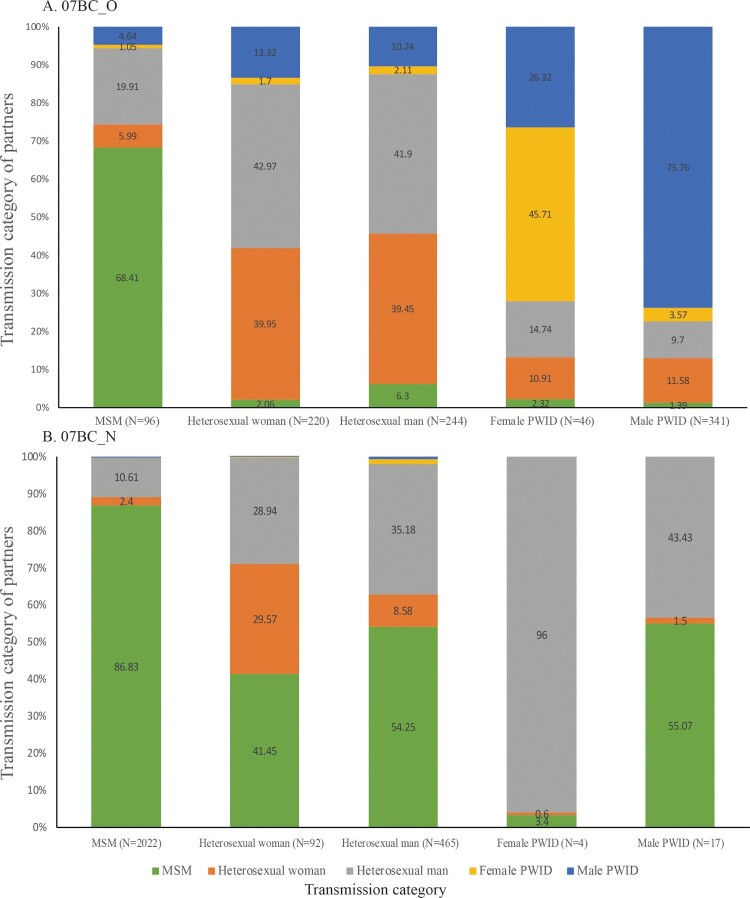
Transmission category of potential transmission partners. This figure shows, for individuals in the transmission network of a given transmission category for 07BC_O (A) and 07BC_N (B), the transmission category of their potential transmission partners. MSM: men who have sex with men; PWID: persons who inject drugs.

Across all risk groups (Figure 6), more than 90% of risk groups except MSM links to PWID and heterosexuals (female and male) within 07BC_O and the proportion of links with PWID and heterosexuals among 07BC_N is evidently lower compared to 07BC_O, but the proportion of links to MSM in 07BC_N showed an uptrend. Only 13.16% MSM were linked to other risk groups within 07BC_N, but these links represented 41.45%, 54.25%, and 55.07% of the links among heterosexual females, heterosexual males and male PWID respectively.

## Discussion

This is the first study using China NCAIDS data to infer a longitudinal HIV-1 CRF07_BC transmission network. We identified two distinct clusters of HIV-1 CRF07_BC strains spreading in China. Since our sequences were collected during 1997–2017, we chose a higher genetic threshold (0.7%) (detailed explanations are available in the supplementary material). The division of the phylogenetic relationships between the two CFR07_BC clusters using transmission networks under the threshold of 0.7% genetic distance was consistent with those inferred using the phylogenetic tree. Each cluster appears to have a unique role in the Chinese HIV-1 CRF07_BC epidemic with distinct associations in particular risk groups and geographic regions. 07BC_O predominantly stems from heterosexual populations, PWID, and married individuals, with most cases reported in northwestern and southwestern China. By contrast, 07BC_N was mainly found in MSM and unmarried individuals among northern provinces. The characteristics of 07BC_N are consistent with previous observations that a large and strongly supported sub-cluster of CRF07_BC strains was observed among young MSM in Jilin, Liaoning, Beijing, and Shijiazhuang (northern provinces) within the CRF07 radiation [7–9,19,20].

Our analysis showed that the major risk cluster for CRF07_BC transmission in China at an early stage was 07BC_O and the first wave of population growth of the CRF07_BC epidemic was driven by 07BC_O. Firstly, 07BC_O was the main cluster in the low and medium-level of TR before 2006. Then, other results showed that individuals, links, density (links/individuals), and the largest cluster of 07BC_O in the cumulative network accounted for the vast majority before 2006. Finally, the Bayesian skyline plot analysis indicates that 07BC_O had undergone significant growth during 1991–2005. Our findings are in line with previous studies that the origin and early epidemic of CRF07_BC in China was in the early 1990s among PWID in Yunnan (southwest) and Xinjiang (northwest) [1,21], and also align with the nationwide HIV-1 molecular epidemiological survey in 2006, which showed that PWID and heterosexuals (07BC_O risk groups) were the primary driver of CRF07_BC transmission [4].

The Bayesian skyline plot analysis indicates the epidemic of 07BC_O had been declining in population size after 2006 and was finally overtaken by 07BC_N. This development coincides with Chinese countrywide policies on universal access to HIV/AIDS prevention, treatment, and care services. In fact, the needle and syringe programme for PWID, free distribution of condoms, and antiretroviral treatment programmes have been rolled out since 2003, and China proclaimed a People’s War on Drugs in 2005 [22]. This led to effective control of transmission among heterosexuals and PWID (07BC_O risk groups) after a few years of the policy implementation.

Further analysis of the longitudinal network revealed that 07BC_N had gradually replaced 07BC_O, and became the major risk cluster for current CRF07_BC transmission in China. TR takes the connectivity of individuals and the number of individuals in each group into account synthetically. A previous study reported that a high degree was more likely to result in a greater risk of HIV-1 transmission [23], and therefore we used TR evaluation for HIV-1 transmission risk of the two clusters. Among populations with high connectivity, 07BC_N was always the majority of the cluster and had continuously increased. The proportion of 07BC_N also increased year by year and exceeded 07BC_O in medium connectivity. These indicate that 07BC_N had a greater risk of HIV-1 transmission compared to 07BC_O. The observed significantly increased odds of clustering and more links in 07BC_N further confirmed this. Then, with the power-law distribution, from the gradually increasing cutoff degree between 07BC_N and 07BC_O, we revealed that 07BC_N not only maintained more distribution than 07BC_O among the population with relatively low connectivity, but also expanded year after year among the population with relatively high connectivity until it was completely over 07BC_O. Furthermore, we also found the similar results that links, density (links/individuals), the largest cluster, and the number of individuals of 07BC_N in the cumulative network have exceeded 07BC_O since 2007, 2007, 2008, and 2010 respectively. Finally, our Bayesian skyline plot analysis indicated that the second period of population growth of CRF07_BC was driven by 07BC_N after 2005. Together, this study is the first study in China indicating that 07BC_N had a greater transmission risk than 07BC_O. The greater risk enabled 07BC_N to gradually overtake 07BC_O, and eventually caused the second wave of the Chinese CRF07_BC epidemic after 2005.

The reason why 07BC_N has a greater transmission risk and spread so rapidly may be associated with the population it circulates in. Previously, we demonstrated that CRF07_BC with relatively lower net charges in the V3 loop exclusively utilizes the CCR5 co-receptor for infection and exhibits slow replication kinetics in the primary target cells. These suggest that CRF07_BC may be superior to other HIV-1 subtypes in initiating infection in high-risk populations in China [24]. This indicates CRF07_BC is more likely popular among high-risk populations (MSM), thus, the resulting independent transmission cluster 07BC_N circulating in high-risk populations have a greater transmission risk. This aligns with Tang’s report that CRF07_BC contained the p6Δ7 mutation dominated in MSM in China [25]. Considering the rapid increase of 07BC_N since 2005, especially after 2010, the rapid increase trend of 07BC_N is consistent with the period of the rapid spread of CRF07_BC among MSM [3,7]. One major reason for this alteration is likely to be the lifestyle changes among MSM. For instance, the use of chemsex was uncommon among Chinese MSM around 2005 [26,27] but has rapidly increased. The use was only 0.8% during 2006–2007 [26], while the proportion rose to 41.8% during 2012–2013 in Beijing, most participants had only begun recently [28]. Prior studies have repeatedly demonstrated significant associations between the use of chemsex and infection with HIV-1 among MSM [29]. The other reason is the popularization of online dating especially among MSM [30]. In 2005, several dating apps and websites such as Renn, blog China, Tianya blog, and QQ Space appeared. Around 2011, WeChat rapidly developed and its users exceeded 100 million by 2012 [31]. Although dating apps and websites are used by many different sub-populations, Grov and colleagues [32] reported that MSM accessed dating apps more frequently than heterosexuals. Furthermore, since the iPhone4 was released in 2010, smartphone use has drastically increased in China. Around 2010, dating apps use on smartphones facilitated MSM to contact with potential partners more conveniently. Before 2005, traditionally conservative Chinese culture made this contact extremely difficult. It was proven that HIV-positive MSM who date online were more likely to have high-risk sexual behaviour and have more sexual partners [33]. Our study indicates that more and more 07BC_N carriers may play the role of super-spreaders through chemsex and online dating to drive the CRF07_BC transmission network in China. The appearance of the super-spreaders is consistent with the rapid spread of CRF07_BC observed over the years [7,34,35] and underscores the importance of focused interventions on 07BC_N.

Our results further suggest that the interventions for the two clusters of CRF07_BC should target various risk groups across the country. More than 90% of risk groups except MSM link to PWID and heterosexuals within 07BC_O, showing that PWID and heterosexuals play a major role in the spread of 07BC_O. The proportion of links with PWID and heterosexuals (female and male) among 07BC_N is lower compared to 07BC_O across all risk groups. This indicates that the needle and syringe programme for PWID and free distribution of condoms for heterosexuals rolled out countrywide in China since 2003 has slowed the spread through injections and heterosexual behaviour. In terms of 07BC_N that has continued to increase rapidly in recent years, we found that a minority of MSM were involved in transmission with other risk groups, including heterosexual males, heterosexual females, and male PWID, but these transmissions represented a substantial proportion of HIV-1 acquisitions by these risk groups. Our finding that MSM became disproportionately connected in the network of 07BC_N implies a concentrated uncontrolled HIV-1 epidemic among MSM. Therefore, this finding underscores the importance of focused interventions on MSM to retard the rapid transmission of 07BC_N because efforts that reduce transmissions involving MSM will not only reduce HIV-1 acquisition in MSM but also lessen HIV-1 acquisition among other risk groups.

In summary, with an unprecedentedly large, longitudinal and detailed HIV-1 CRF07_BC sequence dataset in China, we identified two distinct clusters of HIV-1 CRF07_BC strains spreading in China and revealed that the two clusters are distinct among different risk groups and geographical regions. The first wave of population growth of the CRF07_BC epidemic was driven by 07BC_O before 2005. 07BC_N has a greater risk of transmission, gradually replaced 07BC_O, and caused the second wave of the CRF07_BC epidemic in China after 2005. The divergent transmission risk of the two CRF07_BC clusters highlights the importance of monitoring the genetic evolution and the phenotypic shift at the phylogenetic cluster level to identify and control clusters with greater transmission risk like CRF07_N. We further argue that the rapid increase of 07BC_N since 2005, especially after 2010, was associated with chemsex and online dating. Across CRF07_N, we also revealed that interventions that reduce transmissions involving MSM will also reduce HIV-1 acquisition among other risk groups. This study makes several contributions. First, it helps to grasp the long-term dynamics of CRF07_BC epidemic and factors of CRF07_BC transmission. Second, it provides guidance to target potential risk groups, thus a more effective prevention strategy is proposed to lower HIV-1 CRF07_BC transmission in China.

### Limitations

Limitations of our work open avenues for future research. First, a proportion of our data from LANL may give rise to sample selection bias. Second, some relevant clinical information, such as CD4 count, HIV stage, accurate diagnosis time and the number of sexual partners were not collected. The inclusion of such data in future studies can improve the accuracy of the model to better fit the population distribution.

## Supplementary Material

Supplemental Material
