# Supplementary material for: HIV-1 CRF07_BC transmission dynamics in China: two decades of national molecular surveillance
Source: Emerg Microbes Infect. 2021 Sep 27;10(1):1919–30. doi: 10.1080/22221751.2021.1978822 (PMC8477959; doi:10.1080/22221751.2021.1978822)
Supplement: Supplemental Material [file TEMI_A_1978822_SM7970.docx]

Supplementary Information for

**HIV-1 CRF07_BC Transmission Dynamics in China:** **Two Decades of National Molecular Surveillance**

Zhangwen Ge^a,b,^^c*^, Yi Feng^a*^, Hua Zhang^b^, Abdur Rashid^a,c^, Silvere D. Zaongo^d^,Kang Li^a^, Yueyang Yu^a, c^, Bowen Lv^a^, Jia Sun^a,e^, Yanling Liang^a^, Hui Xing^a^, Anders Sönnerborg^f^, Ping Ma^d^ and Yiming Shao^a,c,e^

^a^State Key Laboratory for Infectious Disease Prevention and Control, National Center for AIDS/STD Control and Prevention, Chinese Center for Disease Control and Prevention, Beijing, People’s Republic of China;

^b^Department of Laboratory Medicine, Guizhou Provincial People’s Hospital, Affiliated Hospital of Guizhou University, Guiyang, People’s Republic of China;

^c^School of Medicine, Nankai University, Tianjin, People’s Republic of China;

^d^Department of Infectious Diseases, Nankai University Second People’s Hospital, Tianjin Second People’s Hospital, Tianjin, People’s Republic of China;

^e^State Key Laboratory for Diagnosis and Treatment of Infectious Diseases, Collaborative Innovation Center for Diagnosis and Treatment of Infectious Diseases, The First Affiliated Hospital, School of Medicine, Zhejiang University, Hangzhou, People’s Republic of China;

^f^Division of Clinical Microbiology, Department of Laboratory Medicine, Karolinska Institutet, Stockholm, Sweden.

Correspondence to: Ping Ma, [mapingtianjin@163.com or](mailto:mapingtianjin@163.com%20or) Yiming Shao, [yshao@bjmu.edu.cn](mailto:yshao@bjmu.edu.cn)

*Zhangwen Ge and Yi Feng contributed equally to this manuscript.

This PDF file includes:

Supplementary Information Text

Supplementary Figures S1, S2 and S3.

Supplementary Table S1

**Supplementary Information Text**

**The Distance Threshold**

Publications that used the HIV-1 molecular transmission network to analyze HIV-1 *pol* sequences have reported a range of genetic thresholds from 0.1% to 2%, with 1.5% being the default and most common value[^1-3^](#_ENREF_1). There are three complementary ways to justify the choice of genetic threshold_._

First, an appealing heuristic approach (exemplified by Wertheim *et al*[^1^](#_ENREF_1)) for tuning genetic threshold would be to select a value that maintains a large number of clusters without creating too many edges. Typically, a range of genetic thresholds around 1-2% emerges as a consequence of this heuristic.

Second, the genetic threshold can be tied to the expected within-host genetic divergence in HIV-1 *pol* during the study duration. There are pragmatic considerations. The genetic threshold in longitudinal and cross-sectional studies are different as HIV-1 mutated over time.

Third, if a particular network-defined quantity is of interest, then one could perform a sensitivity analysis to determine how this quantity depends on the genetic threshold, and a range of values may be chosen so that the inference is not unduly sensitive to the cutoff. There also exist several algorithms, operating on pairwise distances[^4^](#_ENREF_4) or phylogenetic trees^[5](#_ENREF_5)^, that automatically partition data into clusters.

We did a sensitivity analysis on the gene thresholds of 07BC_O and 07BC_N separately (Supplementary Figures S3). When the genetic threshold of 07BC_N was 0.2%, and the genetic threshold of 07BC_O was 0.4%, the transmission network contained the largest number of clusters. Previous studies have shown that a lower genetic threshold can be used to distinguish recent transmissions in outbreak events[^1^](#_ENREF_1) and that a genetic threshold of 0.5% corresponds to being recently infected[^6^](#_ENREF_6), so our genetic threshold should be greater than 0.5%. There is no unified standard for network analysis of long longitudinal collection sequences. Since our sequences were collected during 1997-2017, we should choose a threshold as large as possible to analyze the long longitudinal data. We propose a simple heuristic for deciding on the value of genetic threshold based on the phylogenetic trees and the characteristic of long longitudinal data. The intuitive understanding is as follows. We should choose a higher genetic threshold as the critical threshold. Once the value of genetic threshold exceeds the critical threshold, the network nodes of the two clusters will join one giant cluster, which will no longer provide meaningful information on the underlying epidemiology. The maximum-likelihood phylogeny identified two well supported (bootstrap values>85%) and distinct clusters of CRF07_BC strains, so the genetic threshold was determined based on the results of phylogenetic trees. We analyzed transmission networks under different genetic distances and found that similar observations were made in the transmission network when the maximum genetic distance was 0.7% and CRF07_BC could not be divided into two clusters beyond this threshold. Therefore, we finally chose 0.7% as the genetic distance for our analysis and believed that the threshold used for delineating the transmission networks was reasonable.


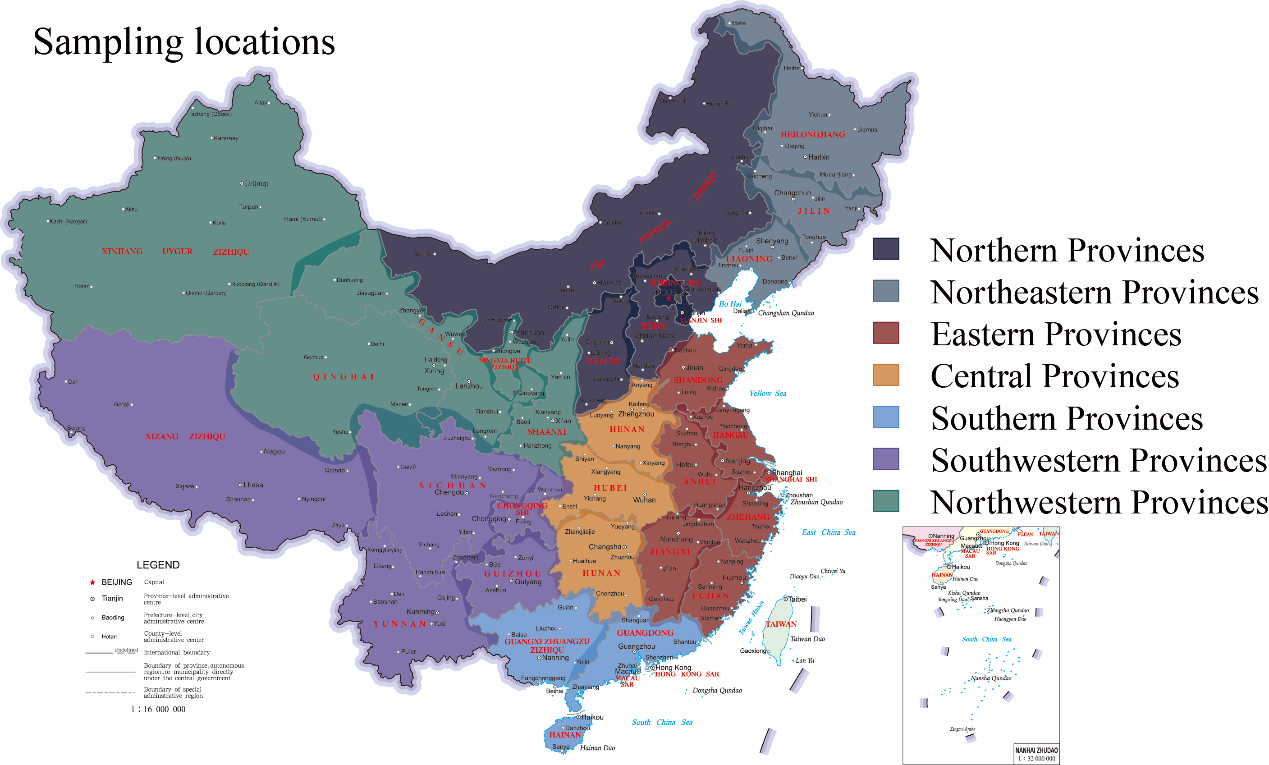


**Supplementary Figure S1. Distribution of sampling locations in China.**

**Supplementary Figure S2:** **Flow chart of the study methods.**

The sequences were extracted from the National Center for AIDS/STD Control and Prevention and Los Alamos HIV-1 sequence database in China before 2017.

Only one sequence (the earliest or the longest) from the same individual was included.

The viral sequences less than 920bp and containing≥5% ambiguities were excluded.

6,213 sequences were collected.

The maximum likelihood phylogenetic tree was built to confirm the genotypes and phylogenetic clusters.

We calculated the genetic distance (TN93) for all pairs of sequences using the HyPhy package.

Determine the value of the genetic threshold based on the phylogenetic trees, sensitivity analysis on the gene thresholds and the characteristic of long longitudinal data.

CRF07_BC was divided into two clusters in the transmission networks when the gene distance was 0.7%: 07BC_O and 07BC_N.

Analyses of the distribution of two clusters in different regions and populations.

Compare two clusters from the number of persons, links, links/persons, and the largest cluster in cumulative transmission works.

The parameter of population transmission risk and power-law function were applied to elucidate the dynamics of both clusters.

Bayesian analysis was used to estimate the epidemic history of two clusters.

Analyses of transmission links between different transmission category.

Analyses of factors associated with clustering


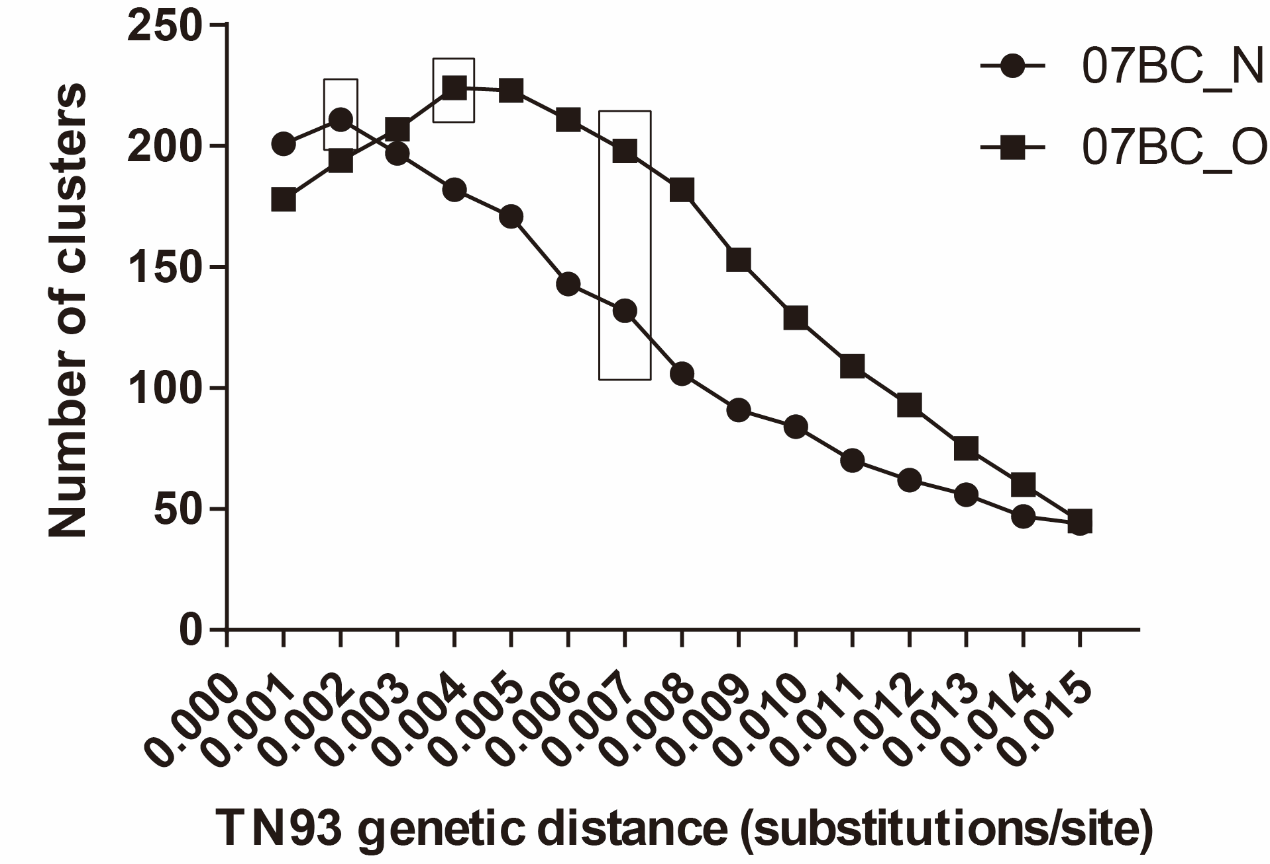


**Supplementary Figure S3. The number of transmission clusters, as a function of the TN93 distance threshold**.

**Supplementary Table S1**. **Factors Associated with Clustering in China Genetic Transmission Network in Logistic Regression Model**

| Attribute | Category | The whole case (n, %) | Case clustered in Network (n, %) | Proportion in Network (%) | OR (95%CI) | *p* Value | ORm(95%CI) | *p* Value |
| --- | --- | --- | --- | --- | --- | --- | --- | --- |
|  | Total | 6213 (100%) | 3607 (100%) | 58.06% |  |  |  |  |
| Cluster | 07BC_O | 2616 (42.12%) | 1000 (27.72%) | 38.23% | Reference |  | Reference |  |
|  | 07BC_N | 3597 (57.89%) | 2607 (72.28%) | 72.48% | 4.29 (3.85,4.78) | <0.001 | 4.37 (3.66,5.22) | <0.001 |
| Sampling Year | 1997-2008 | 535 (8.61%) | 304 (8.43%) | 56.82% | Reference |  | Reference |  |
|  | 2009-2010 | 825 (13.28%) | 595 (16.50%) | 72.12% | 1.97(1.56-2.47) | <0.001 | 1.58 (1.2,2.07) | 0.001 |
|  | 2011-2012 | 977 (15.73%) | 669 (18.55%) | 68.47% | 1.65(1.33-2.05) | <0.001 | 0.7 (0.54,0.92) | 0.009 |
|  | 2013-2014 | 772 (12.43%) | 565 (15.66%) | 73.19% | 2.07(1.64-2.62) | <0.001 | 0.6 (0.45,0.8) | 0.001 |
|  | 2015-2017 | 3104 (49.96%) | 1474 (40.86%) | 47.49% | 0.69(0.57-0.83) | <0.001 | 0.28 (0.22,0.36) | <0.001 |
| Transmission route (birth sex) | Hetero (F) | 844 (13.58%) | 312 (8.65%) | 36.97% | Reference |  | Reference |  |
|  | PWID (F) | 120 (1.93%) | 50 (1.39%) | 41.67% | 1.22 (0.83,1.8) | 0.32 | 0.98 (0.65,1.49) | 0.940 |
|  | PWID (M) | 769 (12.38%) | 358 (9.93%) | 46.55% | 1.49 (1.22,1.81) | <0.001 | 1.17 (0.93,1.46) | 0.171 |
|  | PWID (unknown) | 106 (1.71%) | 40 (1.11%) | 37.74% | 1.03 (0.68,1.57) | 0.877 | 0.41 (0.25,0.66) | <0.001 |
|  | Hetero (unknown) | 42 (0.68%) | 20 (0.55%) | 47.62% | 1.55 (0.83,2.89) | 0.167 | 0.74 (0.38,1.44) | 0.372 |
|  | MSM (M) | 2946 (47.42%) | 2118 (58.72%) | 71.89% | 4.36 (3.71,5.12) | <0.001 | 1.38 (1.11,1.72) | 0.004 |
|  | Hetero (M) | 1386 (22.31%) | 709 (19.66%) | 51.15% | 1.79 (1.5,2.13) | <0.001 | 1.23 (1,1.5) | 0.045 |
| Domicile | Central | 386 (6.21%) | 212 (5.88%) | 54.92% | Reference |  | Reference |  |
|  | North | 1410 (22.69%) | 1011 (28.03%) | 71.7% | 2.08 (1.65,2.62) | <0.001 | 1.36 (1.05,1.76) | 0.020 |
|  | Northeast | 126 (2.03%) | 78 (2.16%) | 61.9% | 1.33 (0.88,2.01) | 0.17 | 1.27 (0.82,1.96) | 0.284 |
|  | Northwest | 662 (10.66%) | 257 (7.13%) | 38.82% | 0.52 (0.4,0.67) | <0.001 | 0.66 (0.49,0.87) | 0.004 |
|  | South | 928 (14.94%) | 684 (18.96%) | 73.71% | 2.3 (1.8,2.95) | <0.001 | 1.11 (0.84,1.48) | 0.462 |
|  | Southwest | 1702 (27.39%) | 720 (19.96%) | 42.3% | 0.6 (0.48,0.75) | <0.001 | 0.91 (0.71,1.17) | 0.455 |
|  | East | 999 (16.08%) | 645 (17.88%) | 64.56% | 1.5 (1.18,1.9) | 0.001 | 0.87 (0.67,1.14) | 0.325 |
| Age at diagnosis | ≤20 | 407 (6.55%) | 239 (6.63%) | 58.72% | Reference |  | Reference |  |
|  | 21-30 | 2359 (37.97%) | 1337 (37.07%) | 56.68% | 0.92 (0.74,1.14) | 0.441 | 1.28 (0.99,1.66) | 0.060 |
|  | 31-40 | 937 (15.08%) | 486 (13.47%) | 51.87% | 0.76 (0.6,0.96) | 0.021 | 0.83 (0.66,1.04) | 0.110 |
|  | 41-50 | 436 (7.02%) | 206 (5.71%) | 47.25% | 0.63 (0.48,0.83) | 0.001 | 0.86 (0.66,1.11) | 0.235 |
|  | 51-60 | 225 (3.62%) | 92 (2.55%) | 40.89% | 0.49 (0.35,0.68) | <0.001 | 0.69 (0.51,0.93) | 0.015 |
|  | >60 | 199 (3.2%) | 92 (2.55%) | 46.23% | 0.6 (0.43,0.85) | 0.004 | 0.75 (0.52,1.09) | 0.137 |
|  | Unknown | 1650 (26.56%) | 1155 (32.02%) | 70% | 1.64 (1.31,2.05) | <0.001 | 1.59 (1.08,2.33) | 0.018 |
| Abrreviations: MSM, men who have sex with men, PWID, persons who use injection drugs, hetero: heterosexual, F: female, M: male; OR: odds ratio was calculated using a univariate logistic regression model, ORm: odds ratio was calculated using a multivariable logistic regression model, CI: confidence interval. The method of the the logistic regression is “step forward multivariable logistic regression”. The dependent variables was “in the network”. | | | | | | | | |
|  |  |  |  |  |  |  |  |  |

**References**

1. Wertheim JO, Kosakovsky Pond SL, Forgione LA, et al. Social and Genetic Networks of HIV-1 Transmission in New York City. *PLoS pathogens* 2017; **13**(1): e1006000.

2. Yuan D, Du Z, Zhou J, et al. HIV-1 subtype diversity, drug resistance, and genetic transmission networks in men who have sex with men with virologic failure in antiretroviral therapy in Sichuan, China, 2011 to 2017. *Medicine* 2019; **98**(43): e17585.

3. Whiteside YO, Song R, Wertheim JO, Oster AM. Molecular analysis allows inference into HIV transmission among young men who have sex with men in the United States. *Aids* 2015; **29**(18): 2517-22.

4. Junqueira DM, Sibisi Z, Wilkinson E, de Oliveira T. Factors influencing HIV-1 phylogenetic clustering. *Current opinion in HIV and AIDS* 2019; **14**(3): 161-72.

5. Feng Y, He X, Hsi JH, et al. The rapidly expanding CRF01_AE epidemic in China is driven by multiple lineages of HIV-1 viruses introduced in the 1990s. *Aids* 2013; **27**(11): 1793-802.

6. Kouyos RD, von Wyl V, Yerly S, et al. Ambiguous nucleotide calls from population-based sequencing of HIV-1 are a marker for viral diversity and the age of infection. *Clin Infect Dis* 2011; **52**(4): 532-9.
